# Relationship between diffusion capacity and small airway abnormality in COPDGene

**DOI:** 10.1186/s12931-019-1237-1

**Published:** 2019-12-02

**Authors:** Rachel N. Criner, Charles R. Hatt, Craig J. Galbán, Ella A. Kazerooni, David A. Lynch, Meredith C. McCormack, Richard Casaburi, Neil R. MacIntyre, Barry J. Make, Fernando J. Martinez, Wassim W. Labaki, Jeffrey L. Curtis, Mei Lan K. Han

**Affiliations:** 1 Division of Pulmonary, Allergy and Critical Care, University of Pennsylvania, 839 West Gates Building, Philadelphia, PA 19104 USA; 2 Imbio, LLC, Minneapolis, MN USA; 3 Department of Radiology, University of Michigan, Ann Arbor, MI USA; 4 Department of Radiology, National Jewish Health, Denver, CO USA; 5 Division of Pulmonary & Critical Care Medicine, Johns Hopkins Medicine, Baltimore, MD USA; 6 Rehabilitation Clinical Trials Center, Los Angeles Biomedical Research Institute at Harbor-UCLA Medical Center, Torrance, CA USA; 7 Division of Pulmonary, Allergy and Critical Care Medicine, Duke University, Durham, NC USA; 8 Division of Pulmonary, Critical Care, and Sleep Medicine, National Jewish, Denver, CO USA; 9 Division of Pulmonary & Critical Care Medicine, Weill Cornell Medical College, New York, NY USA; 10 Division of Pulmonary & Critical Care Medicine, University of Michigan, Ann Arbor, MI USA; 11 VA Ann Arbor Healthcare System, Ann Arbor, MI USA

**Keywords:** Diffusing capacity of the lung (DLCO), Small airways disease (SAD), Chronic obstructive pulmonary disease (COPD), Parametric response mapping (PRM)

## Abstract

**Abstract:**

Impaired single breath carbon monoxide diffusing capacity (DLCO) is associated with emphysema. Small airways disease (SAD) may be a precursor lesion to emphysema, but the relationship between SAD and DLCO is undescribed. We hypothesized that in mild COPD, functional SAD (fSAD) defined by computed tomography (CT) and Parametric Response Mapping methodology would correlate with impaired DLCO. Using data from ever-smokers in the COPDGene cohort, we established that fSAD correlated significantly with lower DLCO among both non-obstructed and GOLD 1–2 subjects. The relationship between DLCO with CT-defined emphysema was present in all GOLD stages, but most prominent in severe disease.

**Trial registration:**

NCT00608764. Registry: COPDGene. Registered 06 February 2008, retrospectively registered.

## Introduction

The importance of small airways disease (SAD) in COPD pathogenesis was first described by Hogg et al. in the 1960s, who determined that peripheral resistance contributes minimally to total airway resistance in healthy lungs but significantly in emphysematous lungs [1]. Recent research using advanced computed tomography (CT) has further suggested that a CT metric for small airway disease (SAD) radiographically precedes emphysema development and is associated with subsequent FEV_1_ decline [2, 3].

Diffusing capacity of the lung (DLCO) is a commonly tested measurement in pulmonary function studies, indirectly measuring degree of gas transfer from alveoli to pulmonary capillary blood, with results depending on the lung’s structural and functional properties. Emphysema irreversibly destroys alveoli, leading to gas exchange impairment and manifesting as an inverse relationship between emphysema severity and DLCO [4]. We hypothesized that CT defined SAD among COPD subjects with mild disease (GOLD 1–2) may detect lung regions in transition from bronchiolar pathology to emphysema, and hence, may be associated with diffusion capacity impairment.

## Methods

We analyzed data from the five-year follow-up visit of the COPDGene cohort in individuals (*n* = 1846) with ≥10 pack-years smoking history and GOLD spirometric grades 0–4 for whom DLCO and CT imaging data were available and obtained on the same day. GOLD 0, although no longer used in the GOLD strategic document, includes non-obstructed smokers (post-bronchodilator FEV_1_/FVC > 0.7). COPDGene is a longitudinal, observational, and multicenter study investigating underlying genetic determinants of COPD (Clinicaltrials.gov identifier NCT00608764). DLCO was measured with EasyOne Pro (serial number 500633, ndd Medizintechnik AG, Zurich, Switzerland). Percent predicted values and z-scores were calculated from raw values using Global Lung Function Initiative (GLI) equations. We processed inspiratory and expiratory CT images by parametric response mapping (PRM), a novel CT biomarker technique, using Imbio Lung Density Analysis dynamic image registration software (Minneapolis, MN) to quantify areas of emphysema (PRM^Emph^) and areas of non-emphysematous gas trapping recently determined to be SAD (PRM^fSAD^) [5]. Multivariable regression models for DLCO GLI z-score were created to determine the relative contribution of PRM^Emph^ and PRM^fSAD^, additionally adjusted for age, sex, and current smoking.

## Results

Participant mean age at time of measurement was 67.0 years. With increasing GOLD stage, DLCO % predicted fell and z-scores became more negative, while PRM^Emph^ and PRM^fSAD^ rose. These characteristics, as well as other subject characteristics, are shown in Table 1.

**Table 1:** Baseline demographics

	GOLD spirometry grade		
	0 *(n = 957)*	1–2 *(n = 584)*	3–4 *(n = 305)*
Age, yr	63.5	68.3	68.9
Sex, n (% female)	512 (54%)	249 (42%)	130 (43%)
BMI (kg/m^2^)	28.9	27.9	27.5
Smoking pack-years	37.1	49.8	55.0
Current smokers, n (%)	344 (36%)	212 (36%)	61 (20%)
Exacerbations in prior year	0.11	0.32	0.71
FEV_1_, L	2.73	2.03	0.98
FEV_1_% predicted	98.4	73.3	36.4
FVC, L	3.49	3.39	2.43
FVC % predicted	96.1	91.7	67.2
FEV_1_/FVC	0.78	0.60	0.41
DLCO, % predicted	90.1%	74.2%	51.2%
DLCO, GLI z-score	−0.73	−1.91	−4.08
PRM^fSAD^	10.0%	21.0%	34.6%
PRM^Emph^	1.8%	6.5%	18.3%

All values expressed as mean except categorical variables expressed as n (%)

*BMI* body mass index, *FEV*_*1*_ forced expiratory volume in 1 s, *FVC* forced vital capacity, *DLCO* single breath carbon monoxide diffusing capacity of the lung, *GLI* Global Lung Function Initiative, *PRM* parametric response mapping, *fSAD* functional small airways disease, *Emph* emphysema

In a multivariable regression model examining non-obstructed ever-smokers, PRM^fSAD^ (β = − 0.03, *p* < 0.001) and PRM^Emph^ (β = − 0.04, *p* = 0.03) were associated with lower DLCO GLI z-score. For clinical interpretation, on average a non-obstructed individual with 10% higher PRM^fSAD^ would be predicted to have 3.1% lower DLCO % predicted. In GOLD 1–2, both PRM^fSAD^ (β = − 0.02, *p* = 0.004) and PRM^Emph^ (β = −.10, *p* < 0.008) were associated with lower DLCO GLI z-score. Among GOLD 3–4, PRM^Emph^ (β = − 0.11, *p* < 0.001) but not PRM^fSAD^ (*p* = 0.69) was associated with lower GLI z-score.

## Discussion

Our analysis of a sizeable group of current and former smokers indicate that in those without airflow obstruction and in individuals with mild to moderate COPD, small airways disease defined by PRM is associated with significant gas exchange abnormalities. These data build on and extend recent work to elucidate the nature of small airway abnormality in COPD. The PRM^fSAD^ metric has been associated with more rapid lung function decline, even among individuals with emphysema [6]. Using longitudinal image registration, we previously showed that voxels identified as PRM^fSAD^ radiographically precede development of CT-determined emphysema in those same voxels [7]. Importantly, we recently demonstrated with severe COPD human lung tissue that PRM^fSAD^ metric corresponds to pathologic abnormality, including decreased circularity, decreased luminal area, and complete obstruction of terminal bronchioles (TBr), whereas PRM^Emph^ is significantly associated with decreased alveolar surface area [5].

However, the pathology identified by PRM^fSAD^ in milder disease is unknown. Given the totality of data generated to date, PRM^fSAD^ is also likely associated with TBr pathology in mild COPD, but the extent to which it may also identify early alveolar destruction must be determined.

The association we show here between PRM^fSAD^ and reduced DLCO suggests that the fSAD metric may detect lung beginning to transition from bronchiolar pathology to emphysema.

Support for this possibility comes from McDonough et al., who used micro-CT to analyze lung tissue samples in severe COPD. By identifying significantly reduced TBr numbers in areas of lung without visible emphysema evidence, they inferred that narrowing and loss of TBr precedes emphysema [2]. However, a linear relationship between loss of TBr and increase in mean linear intercept (a measure of mean free distance in the air spaces) was seen. Most recently, Koo et al. examined lung tissue from GOLD 1–2 stage disease and again found significant reductions in number of TBr and transitional bronchioles in lung areas without visible emphysema [3]. Collectively, these results support SAD as one precursor of emphysema.

DLCO is well-established to correlate with CT-determined emphysema [4], but it may also be decreased in mild COPD, where emphysema is not detectable by CT imaging. Active smokers with normal spirometry but low DLCO had increased rate of progression to GOLD-defined COPD, compared to smokers with normal spirometry and normal DLCO, implying that isolated decreased DLCO is a risk factor for airflow obstruction in otherwise healthy subjects [8]. Ex-smokers with normal CTs and no airflow obstruction but with low DLCO had significantly worse symptoms and exercise capacity as well as greater regional lung destruction, as measured by hyperpolarized ^3^He MRI apparent diffusion coefficients [9]. In 10 patients with severe SAD, defined by severe expiratory airflow limitation with mild CT-detected emphysema, Gelb et al. found DLCO reductions; however, they proposed that this was possibly due to uneven gas sampling from lung units with differing expiratory time constants, a function of SAD rather than lung parenchymal destruction [10].

Our current analysis of ever-smokers without obstruction and with GOLD 1–2 COPD showed that PRM^fSAD^ correlates with low DLCO, suggesting PRM^fSAD^ might detect airways transitioning to early emphysema with resulting impaired gas exchange. Although COPD is a heterogonous disease, it is possible that a significant portion of small airways disease patients will progress to emphysema. Therefore, in addition to already describing correlations between tissue pathology and PRM^fSAD^ in severe disease, similar studies in milder disease are also needed to confirm the pathology type identified in this population, who may be more amenable to therapeutic intervention that may halt progression to emphysema.

## Data Availability

The datasets used and analyzed during the current study are available from the corresponding author on reasonable request.

## Abbreviations

CT: Computed tomography
DLCO: Diffusing capacity of the lung
fSAD: Functional small airways disease
GLI: Global Lung Function Initiative
PRM: Parametric response mapping
PRM^Emph^: Emphysema as determined by PRM
PRM^fSAD^: Small airways disease as determined by PRM
SAD: Small airways disease
TBr: Terminal bronchioles
